# A qualitative analysis of parents’ beliefs about portable pool safety behaviours

**DOI:** 10.1177/13591053241275588

**Published:** 2024-09-18

**Authors:** Kyra Hamilton, Kim Dunn, Jacob J Keech, Amy E Peden

**Affiliations:** 1 Griffith University, Australia; 2 University of Jyväskylä, Finland; 3 University of California, Merced, USA; 4 University of New South Wales, Australia; 5 James Cook University, Australia

**Keywords:** beliefs, children, family, health behaviour, qualitative methods, theory of planned behaviour

## Abstract

The aim of this study was to develop an in-depth understanding of the beliefs parents hold regarding portable pool safety behaviours using the theory of planned behaviour (TPB). Semi-structured interviews were conducted with parents (*N* = 15) of children aged 5 years and younger who owned a portable pool. Interviews examined three key safety behaviours: supervising within arms’ reach, fencing portable pools deeper than 30 cm, and emptying and storing portable pools safely after use. Parents identified a range of advantages, disadvantages, normative influences, and facilitators and barriers towards the three behaviours. The identification of these salient behavioural, normative, and control beliefs enrich limited understandings of portable pool safety behaviours of parents with young children. Current findings fill a knowledge gap in portable pool safety and provide potential targets for messages to improve parents’ behaviours for their young children around portable pools in the hope of preventing loss of life.

## Introduction

Globally, children and young people under 25 years represent the majority of deaths due to drowning (World Health Organization, 2014). In Australia, unintentional drowning is a significant cause of premature mortality and morbidity for young children (Australian Institute of Health and Welfare, 2023). Beyond fatalities, non-fatal drowning represents a significant cause of hospital admission for children under five, with studies indicating for every fatality there are eight children admitted to hospital for a drowning incident (Peden et al., 2018).

Water bodies in and around the home environment, in particular swimming pools, pose the greatest drowning risk to young children. Within the swimming pool category, portable pools (inflatable, flexible canvas, or plastic attached to a frame or hard plastic and non-permanent pools) (Royal Life Saving Society – Australia [RLSSA], n.d.) are a location of concern given their accessibility, being widely available for immediate purchase in store, and at substantially lower cost than a large backyard pool. Social determinants of health influence drowning deaths of young children in portable pools, with children residing in socio-economically disadvantaged areas and in regional and remote locations disproportionately affected (Peden et al., 2020).

Given child drowning concerns, the #MakeItSafe portable pool safety campaign, was developed by drowning prevention and consumer safety authorities (Consumer Protection Western Australia, n.d.). #MakeItSafe focuses on raising awareness among parents and caregivers of evidence-based child drowning prevention strategies, such as adult supervision, emptying and storing portable pools after use, and learning cardiopulmonary resuscitation. In addition to these, the campaign promotes the legislative requirement (in almost all Australian states and territories) to fence any pool 30 cm or more in depth, although this requirement is poorly understood and rarely enforced (Peden et al., 2020). Similarly, while parent and caregiver awareness of child drowning prevention strategies that can be employed for private swimming pools are having an impact on child drowning rates (Peden et al., 2021), awareness of the dangers of portable pools are significantly lower. Thus, there is a need to better understand parent and caregiver knowledge of drowning prevention interventions specific to portable pools, and the beliefs they hold that may guide their decisions to comply with portable pool safety behaviours.

The theory of planned behaviour (TPB; (Ajzen, 1991)) is a well-used model applied to the understanding of social, health, and safety behaviours (Hagger and Hamilton, 2024). The TPB proposes that intention is the most proximal predictor of behaviour, with three main belief-based social cognition factors proposed to influence intention: attitude (expected outcomes of the behaviour), subjective norm (pressure from significant others to do the behaviour), and perceived behavioural control (control and capacity over behavioural action). Underlying these three global determinants are sets of more specific corresponding salient beliefs; behavioural beliefs (advantages/disadvantages), normative beliefs (approval/disapproval), and control beliefs (facilitators/barriers) (Ajzen, 1991), which provide insights for targeted messaging.

Studies have used the TPB belief-based framework to understand the safety beliefs parents hold when making decisions for their child (Hamilton et al., 2020), including for drowning prevention behaviours (Hamilton et al., 2019; White et al., 2018). The TPB belief-based framework has also been used successfully to design messages and programs to change people’s behaviour around water (Hamilton et al., 2021, 2022). It therefore seems useful to apply a TPB belief-based framework to understand parents’ portable pool safety behaviours (i.e. supervision, ensuring fencing is in place, and emptying and storing portable pools after use). Such knowledge may allow the development of theoretically-based and empirically-driven behaviour change initiatives that are pertinent to strategies for reducing childhood drowning deaths in portable pools (Ajzen, 2015). The aim of the current study was to explore parents’ behavioural, normative, and control beliefs about portable pool safety behaviours utilising a TPB belief-based framework within a qualitative approach.

## Methods

### Research design

A qualitative research design was employed, adopting a semi-structured interview method together with a theoretical thematic analysis approach (Braun and Clarke, 2013). Thematic analysis focuses on examining patterns and or themes of meaning within data (Braun and Clarke, 2013), and in the current context helps to provide a rich understanding of beliefs and behaviours of parents who own a portable pool.

### Sampling and recruitment of participants

A purposive sampling method (Patton, 2002) was adopted to recruit Australian parents 18 years and older, and who had at least one child aged 5 years and younger, and who owned and used a portable pool. Purposive sampling allowed for a range of parents with varying geographic locations to be recruited and ensure a range of information-rich cases and beliefs were captured. Participants were recruited using email and social media notices, and snowballing. Participants were primarily community members from metropolitan areas of Brisbane and the Gold Coast, Queensland, Australia. All participants who provided availability to be interviewed were interviewed, and none withdrew from participating. Ethics approval was granted by the University Human Research Ethics Committee (2018/951).

### Sample size

Participants (*N* = 15) were aged between 21 and 42 years (*M_age_* = 33.47 years, *SD* = 5.80), with the majority being female (*n* = 11). All except one participant came from an English-speaking background (*n* = 13), and one unknown. Whilst all participants had children under 5 years of age, six had more than one child under the age of 5 years. The children’s age ranged between 5 months to 5 years (*M_age_* = 2.6 years, *SD* = 1.35) with the majority of children being female (*n* = 14). Education levels varied among participants; postgraduate degree (*n* = 4), undergraduate degree (*n* = 6), vocational qualification (*n* = 3), senior certificate (*n* = 1), and junior certificate (*n* = 1). Marital status reported by participants was married (*n* = 11), single (*n* = 2), and separated or divorced (*n* = 2). Most of the participants were engaged in various kinds of employment, whilst one participant was on maternity leave, and one was unemployed. Participants were offered a payment of AU$50 for their contribution to the study.

### Study rigour

We applied reporting guidelines as set out in the consolidated criteria for reporting qualitative research (COREQ) to ensure thoroughness and creditability (Tong et al., 2007). The COREQ is a 32-item checklist (refer to Supplementary File 1) intended to improve the standard of reporting for qualitative research across three domains: research team and reflexivity, study design, and analysis and findings (Tong et al., 2007).

### Research team and reflexivity

The research team consisted of university-based researchers (KH, KD, and JK) and an industry-based researcher (AP) with knowledge in drowning prevention, health promotion, behavioural psychology, as well as qualitative research. Reflexivity in qualitative research is important due to the subjective nature of qualitative data and methodology. Reflexivity practices in this study were achieved by keeping a reflexive journal (a process whereby researchers document their own reflections and thoughts during data collection) throughout the research process as well as keeping an open dialogue and discussion among all team members to identify and account for any subjective bias of the researchers.

### Data collection

Prior to participation, parents were provided with an information sheet describing the study and were given the opportunity to ask questions. After providing verbal consent, participants completed a short demographic survey. Semi-structured interviews were conducted via telephone and were approximately one hour in length. The interviewer (JK, male, PhD, employed as a research fellow) held a reflexive journal in which ideas, appraisals, and refinement of questions were noted (Braun and Clarke, 2006). Interviews were audio-recorded and transcribed verbatim, and then analysed in NVivo (NVivo, Version 12.0, 2019). Author KD (female, BS (Honours), employed as a medical laboratory assistant) coded the interviews. Author JK co-coded 10% of transcripts. Codes (captured as a single belief associated with a segment of the data) were then organised into themes (captured as common, reoccurring patterns of beliefs across the data set) by author KD. Themes were discussed among the research team prior to being finalised.

### Measures

The target behaviours were three key portable pool safety behaviours that are advocated to reduce drowning (Consumer Protection Western Australia, n.d.); (1) *supervising within arm’s reach;*(2) *fencing the portable pool deeper than 30* *cm* and (3) *emptying and storing the portable pool safely after use.* To investigate these specific behaviours, an interview guide and protocol was developed by the research team. The survey and interview questions (refer to Supplementary File 2) were developed to gather important demographic data and stimulate meaningful conversation about parents’ beliefs towards the portable pool safety behaviours.

After the short demographic survey, the interview comprised of four sections. The first section enquired about parents’ knowledge of portable pools, the type of portable pool they own, their understanding of the legal responsibilities as portable pool owners, and how they perceive the drowning risk around a permanent in-ground pool compares to the drowning risk of a portable pool for young children. The next three sections targeted the three key safety behaviours to elicit parents’ beliefs about portable pool safety behaviours and is the focus of this study. The open-ended questions were designed according to TPB guiding principles proposed by Ajzen (2006) and were aimed at eliciting behavioural beliefs, normative beliefs, and control beliefs for each of the key safety behaviours (Supplementary File 2). Upon conclusion of the interview, participants were asked to share any further input about portable pool use they believe is valuable to the area of research. To ensure the accuracy in data interpretation, confirming summaries were performed throughout the interview. No repeat interviews were conducted, nor were transcripts returned to participants.

### Analytic strategy

Theoretical thematic analysis was used to explore patterns in the data and report themes that were identified (Braun and Clarke, 2013). The interview data was analysed according to the six phases proposed by Braun and Clarke (2013). This method was preferred as it allows data driven themes to be organised based on theoretical concepts and existing theory. Phase 1 was data familiarisation, through transcribing the interviews verbatim, thoroughly reading each transcript, and observing patterns and meanings pertinent to the research questions. Phase 2 involved initial coding of the data. On completion a code-recode procedure was performed for 10% of the data to ensure stability and consistency of coding. Phase 3 involved searching for patterns that were theoretically meaningful and organising them into themes. In Phase 4 the themes were revised and refined, confirming the themes accurately captured the correct meanings. Phase 5 defined and named each overarching theme. Phase 6 involved producing the report of themes and providing a selection of rich interview quotes.

## Results

Participants’ accounts and the subsequent beliefs they described as underpinning their portable pool safety behaviours (supervising within arms’ reach, fencing portable pools deeper than 30 cm, and emptying and storing the portable pool safely after use) are presented below. Given that the study aim was to explore the most frequently held parental beliefs about portable pool safety behaviours, only the most salient themes (groupings of similar beliefs) are presented. The following themes were developed by the researchers from data collected from parents’ own descriptions and experiences of portable pool ownership and associated safety behaviours, ground in the TPB belief-based framework. See Supplementary File 3 for a summary of key themes and supporting quotes of behavioural, normative, and control beliefs. Participant number has been used to classify quotes to ensure confidentiality (e.g. *P12*). Supplementary Figures 1 to 3 depict thematic maps for the three target behaviours.

## Target behaviour 1: Supervising within arm’s reach (Supplementary Figure 1)

### Behavioural beliefs: Advantages and disadvantages of supervising within arm’s reach

#### Advantages

##### Quick response to prevent incident or injury

The most commonly described advantage by parents was the ability to react swiftly to any adverse situations should they arise. The quick ability to negate any injury or risk of drowning when staying within arm’s reach was described by most parents.

##### Engagement and bonding

Supervision within arm’s reach for many parents was described as a time to spend with your child, engaging in play activities with them. It was further described by several parents as an opportunistic time to spend bonding since they are sitting there supervising anyway.

##### Educational

Several parents described using supervision as an opportunity to teach water safety concepts (e.g. kicking and paddling skills) and appropriate behaviours while in and around the pool (e.g. correcting unsafe behaviours such as climbing or jumping).

##### Child’s sense of security

Many described the presence of an adult within arm’s reach creates a sense of security for the child, enabling them to feel confident in what may be described as an unfamiliar environment for some children.

#### Disadvantages

##### Can’t do anything else while supervising and it can be a boring task

The most commonly described disadvantage by parents was nothing else gets achieved whilst supervising. Comparing it to a menial task that sometimes, as a parent, they may not want to do.

##### Creates a lack of independence and confidence

For many parents, ‘hovering’ too closely was discussed as potentially hindering the child’s ability to learn independence and responsibility. Also, that the child might become reliant on the parent if they are always present to prevent them from going face down in the water.

##### Having other children

Having more than one child to supervise was described as a barrier, as this presented a dilemma for the parent in deciding where their attention should be directed.

### Normative Beliefs: approval and disapproval of supervision within arm’s reach

#### Approve

##### Water safety advocates

Majority of parents described water safety advocate groups such as surf lifesaving and Laurie Lawrence (ex-Australian swim coach who leads a child drowning prevention campaign called Kids Alive Do the Five), as supporting supervision within arm’s reach. Largely parents were able to draw on knowledge of water safety groups and the idea of these groups promoting water awareness.

##### Family

Family members, particularly parents and significant others, were identified as supporting their decision to supervise within arm’s reach. Describing that their loved ones would expect them to look after their children appropriately.

##### Friends

For some parents, it was also their friends that were supportive of supervising within arm’s reach. That their ‘circle of friends’ who also have their own children, hold similar beliefs around the importance of supervision within arm’s reach.

##### Council, government, and health agencies

Many parents described various government agencies including health workers, police, and child safety agencies as supporting their decision to supervise, especially those who have observed the consequences of children who have not been supervised.

##### Other parents

Some participants described other parents as approving. Stating that other mums would want supervision to take place if their child was with you.

#### Disapprove

##### No one would disapprove

For the majority of participants, no one came to mind who would think negatively towards the behaviour.

##### Older generations

Some parents described the older generation as disapproving of such close supervision. Grandparents that might think you’re ‘mollycoddling’ or ‘helicopter parenting’ your child, rather than just letting them ‘sink or swim’. The ‘old fashioned’ attitude that older generations may possess of being less conscientious about supervising children.

### Control Beliefs: facilitators and barriers of supervision within arm’s reach

#### Facilitators

##### Comfortable location

Being in a comfortable, shady location with a chair was the most commonly described facilitator of supervision. Also described was having a clear view so there were no obstacles or obstructions hindering one’s view; yet also staying out of splash distance.

##### Being prepared and organised

Followed by being prepared, making sure that you are organised with towels, water, hats, and snacks was a facilitator. Having everything close by that is required was recognised by parents as important to not divert attention away from supervising.

##### Having no distractions

Having no distractions was described by some parents as a facilitator of supervising their children. Having their attention directly on their child in the pool was an important part of supervising.

##### Dedicating time

Dedicating time, specifically for the activity and not scheduling or trying to do other things at the same time, was described as making it easier for supervision. One parent described it as setting aside specific times during their day to let their child play in the pool.

##### Having another adult to help

Also noteworthy was that parents described having another adult who is able to step in and take over supervising to allow the parent to complete other tasks as a facilitator. Furthermore, it was described as being ‘more enjoyable’ having another adult to talk to and keep them company.

#### Barriers

##### Distractions

Most parents described distractions as the main barrier to supervising within arm’s reach. Identifying social media and phone use as the most commonly reported distraction. Also, domestic situations such as someone arriving at your front door and having other children to attend to were noted as barriers.

##### Not being prepared or organised

Not being organised and prepared was identified as the opposite of what parents were describing as facilitators. Not being organised and prepared for the activity was seen as a distraction.

##### Child factors

Child factors such as age and developmental stages were described as another barrier to supervision. It was described that at a younger age, children can be quite ‘impulsive’ and ‘hard to rationalise’ with.

##### Not having the space, size, and location

For some parents, not having enough space for both the pool, the child, and the parent to occupy was a barrier. If the pool is too large or in a difficult location, it is harder to be within arm’s reach of the child without getting in.

## Target behaviour 2: Fencing portable pools deeper than 30 cm (Supplementary Figure 2)

### Behavioural beliefs: Advantages and disadvantages of fencing portable pools

#### Advantages

##### Prevention of drowning and safety

The most commonly described advantage of fencing a portable pool was that it would prevent drownings and stop children from entering the pool unknowingly, essentially negating the potential drowning risk for any children in the area.

##### Peace of mind

For most parents, having peace of mind that they can walk away from their child and do other things and not have to worry was an advantage to fencing, believing the notion that their child will be safe and kept separated from the pool.

#### Disadvantages

##### Expensive and costly

The most commonly identified disadvantage to fencing a portable pool was the expense. Pool fencing was described as an expensive item to buy for an inexpensive portable pool that was only going to be used for short periods of time, asserting that the pool fence would be worth considerably more than the pool itself.

##### Becomes a permanent fixture

Many parents described portable pool fencing as defeating the purpose of it being ‘portable’. Making a portable item a permanent fixture, limiting its uses and contradicting its actual purpose.

##### Creates additional hassles

Some parents identified portable pool fencing as creating more hassles. Referring to things like council permits and inspection requirements as an annoyance. Also noteworthy was that pool fencing makes it harder for the parent to access the pool area especially when their hands were full.

### Normative beliefs: Social approval and disapproval of fencing portable pools

#### Approve

##### Council, government, and health agencies

Similar to supervision, many parents described various government agencies including health workers, police, and child safety agencies as approving the installation of fencing.

##### Family

Family members, in particular parents and partners, were among the individuals that parents described as approving, asserting that their family would not want anything to happen to their loved ones by accident.

##### Friends

For some parents, it was their friends that were believed to be supportive of installing pool fencing, although most participants did not elaborate on why they held this belief.

##### Majority of people

For some parents, they simply described that most people would approve. This included the wider community, especially if the portable pool was located in a front yard where it could be easily accessed by children.

##### Neighbours

Neighbours were also identified as a group that would approve of fencing. Describing that it is not only their own children they have to consider, but the safety of others that live nearby.

##### Water safety advocates

Water safety advocates were identified as groups that would approve of fencing. Specifically, lifesavers and Laurie Lawrence (a prominent drowning prevention educator and advocate) were mentioned as approving of the behaviour.

#### Disapprove

##### Landlords

For parents who rented, landlords were identified as a group that would most likely disapprove. Making structural changes to a rental property was viewed as not being allowed without approval from the landlord.

##### No one would disapprove

Some parents reported not being able to identify anyone who would disapprove of fencing a portable pool.

### Control beliefs: Facilitators and barriers of fencing portable pools

#### Facilitators

##### Clear guidelines, awareness, and education

The most identified facilitator of installing a pool fence was having clear guidelines about what constitutes a pool fence, understanding the law’s requirements and where to purchase a fence.

##### Make cost effective or subsidise

For many parents, if the cost of fencing was affordable or even subsidised partly, it would facilitate the capacity to fence their portable pool. One parent described the cost as a ‘huge thing’ when it comes to fencing a portable pool.

##### Package deal; pool and fence sold together

A few described having a ‘package deal’ where the pool and the fence are sold together would help facilitate fencing behaviours and eliminate extra work that is involved (e.g. sourcing fencing).

#### Barriers

##### Difficult and a hassle

For the majority of parents, fencing was described as creating more of a hassle then just having the pool itself, and they would be less likely to own one if they had to fence it.

##### Unclear requirements and lack of knowledge

The ambiguity of what constitutes an adequate pool fence, together with the lack of knowledge and skill to install a pool fence, was described by many participants as a substantive barrier.

##### Cost

For many parents, simply the expense of fencing was considered a barrier. It was likened to a ‘financial burden’ by one parent and not a ‘cheap exercise’ by another.

##### Rental properties

For parents living in rental properties the disadvantage that was described was not having the ability to add fencing structures to facilitate the requirements.

##### Space to erect a fence

For some parents, not having adequate space to erect a fence was a disadvantage, implying that smaller properties may not physically have the space to install a fence.

## Target behaviour 3: Emptying and storing portable pools safely after use (Supplementary Figure 3)

### Behavioural beliefs: Advantages and disadvantages of emptying and storing the portable pool safely

#### Advantages

##### Prevents drowning and ensures safety

The most commonly described advantage was how the drowning risk is negated when the pool is emptied and stored after use. Also notable was the way parents felt it was a ‘weight off’ their mind knowing it was packed away safely.

##### Prevents mould, algae, and bacteria, and maintains water hygiene

For most parents, the ‘cleanliness’ of the water was imperative. Emptying the pool after use would prevent the growth of algae and waterborne diseases and becoming ‘gross’ and ‘festy, diseased and disgusting’.

##### Prevents mosquitos

The prevention of mosquito breeding was identified as an advantage. One parent mentioned the importance of emptying to prevent breeding which is a risk due to her location.

##### Preserves the life of the pool

For some parents, the idea of emptying and storing their pool was a way to preserve the life of the pool and will prevent the pool from disintegrating, reporting that looking after it will make it last longer.

##### Reuse the water to feed gardens and lawns

Recycling the water and using it to water the lawns and gardens was a consideration for some parent. Also describing that they feel they are ‘doing their bit’ for water conservation.

#### Disadvantages

##### Time and effort

For the majority of parents, it was the time and effort involved in the emptying, drying, and deflating process that was described as a disadvantage to performing the behaviour. Also mentioned was the effort of having to go through a similar process; inflating and refilling then emptying and storing again.

##### Excess water use and cost

The excess water and costs associated with the emptying and refilling process was identified as a considerable disadvantage. Also mentioned was the environmental impact in drought affected areas.

##### The size and design of the pool

The size of the pool was influential to the target behaviour, implying that the bigger the pool the more disadvantaged the process becomes. The larger the pool, the heavier it is, and harder it is to empty.

### Normative beliefs: Approve and disapprove of emptying and storing the portable pool safely

#### Approve

##### Family

The most commonly reported group that would approve was family. With partners being described the most often, followed by parents. The expectation that their family would want them to perform the target behaviour to ensure the children are kept safe.

##### Council, government, and health agencies

For some parents, water and health organisations were considered to approve of emptying and storing the pool to minimise stagnant water and potential for waterborne diseases from growing. Further, to negate the risk of immersion injuries that an unemptied pool may yield.

##### Water safety advocates

A commonly describe group that would approve was that of water safety advocates (e.g. lifesavers and Laurie Lawrence) who would want to keep drowning risk as low as possible by emptying and storing the pool safely.

##### Everyone and anyone

When asked for groups that would approve, some parents simply described everyone as approving of the behaviour. One parent couldn’t understand why you would want to leave a body of water around, likening it to a ‘ticking time bomb’.

##### Friends

For some parents, friends were believed to approve of emptying and storing the pool, stating that their friends would expect that their pool would be emptied and stored and not sitting around getting filthy.

#### Disapprove

##### Water authorities and drought affected people

Drought affected people and environmentally friendly individuals were considered as not approving of the excess water usage that is associated with performing this behaviour. Also identifying water authorities and councils as not approving whilst water restrictions were in place.

##### No one would disapprove

For some participants, no one came to mind who would feel negatively towards the behaviour, with no further elaboration on their responses.

### Control beliefs: Facilitators and barriers of emptying and storing the portable pool safely

#### Facilitators

##### Ease of draining, deflating, and drying

The factors that make it easy to drain, deflate, and dry was the most commonly identified facilitator of the target behaviour. Parents described having the drain plug in a better location for easier draining of the pool. Also mentioned was having a quick release air valve to accelerate the deflating process.

##### Size and design of the pool

For some parents, smaller designed portable pools were considered easier to empty and store. Identifying that a smaller pool is more manageable and lightweight as opposed to a larger inflatable pool that can be quite ‘cumbersome’.

##### Space to store it

For other parents, having enough space to store the pool was described as making it easier to empty and store regularly.

#### Barriers

##### Time consuming

For most parents, the most salient barrier to emptying and storing was time. The timely process of draining the pool, then deflating it and ensuring the pool is dried before storing the pool to prevent mould.

##### Slow to drain

Some parents described the slow draining of the pool or the lack of a drain plug to assist with the emptying the water from the pool as a barrier to performing the behaviour.

##### Heavy weight

The heavy weight of the pool was also reported as a barrier to emptying the pool. One parent stated it was the sheer size of the pool that makes it difficult to empty and store. While another called it ‘bulky and cumbersome’.

##### No barriers

Some parents, however, simply reported there were no barriers to emptying and storing the pool, with one parent stating that ‘nothing is really hard about it’.

## Discussion

While research exploring parents’ backyard pool safety behaviours had been established (Hamilton et al., 2019; White et al., 2018), there is a dearth of research exploring the beliefs of parents towards portable pool safety behaviours. The current study fills this knowledge gap, adopting qualitative methods and using a TPB belief-based approach to gain a rich understanding of salient behavioural, normative, and control beliefs guiding parents’ decisions to supervise around, properly fence, and safely empty and store their portable pool. Results may inform strategies for safety messages around portable pools at the individual level but could also support development of community-level initiatives targeting different social ecological levels of water safety (McLeroy et al., 1988).

### Advantages: Reduce drowning risk, child engagement, and health benefits

Behavioural beliefs are framed by potential advantages and disadvantages and inform attitudes which have been shown to predict backyard pool safety behaviours (Hamilton et al., 2019; White, 2018). The most commonly described advantage across all three target behaviours was the reduction of drowning risk. This suggests that parents are taking note of the benefits of performing these behaviours and that safety messages are reaching Australian parents. Beyond this, engagement and bonding of the parent and child, and educational opportunities surrounding water safety were further advantages reported of supervising within arm’s reach. In line with longstanding research regarding parental behaviours more broadly (Hoover-Dempsey and Sandler, 1995; Salter Ainsworth and Bell, 1981), drowning prevention and consumer safety organisations should highlight the additional child development benefits, beyond drowning prevention, of portable pool safety behaviours. Similarly, the more unique, yet practical health benefits identified by parents associated with emptying and storing the portable pool safely after use (i.e. the prevention of bacteria, mould and mosquitos for better pool hygiene, preservation of the pool and recycling of the pool water) future portable pool safety interventions could also address these beliefs.

### Disadvantages: Inability to multi-task, limits child’s independence, fencing costs, and maintenance

Disadvantages were also discussed. The most commonly described disadvantage by parents was that nothing else gets achieved whilst supervising and comparing it to a menial task that sometimes, as a parent, they may not want to do. Additionally, another disadvantage for the behaviour of supervising within arm’s reach was the notion of creating an inability for the child to learn independence and responsibility and build confidence. Paradoxically, as parents identified supervision within arm’s reach as ‘hovering’ too closely as a hindrance to a child’s development, the closeness of the parent supervising within arm’s reach was also identified as an advantage, providing the opportunity for engagement and bonding and, therefore, providing a sense of security for the child. This attitudinal ambivalence (i.e. holding both positive and negative appraisals) (Schneider and Schwarz, 2017) may have an attenuation effect on the decisions of parents towards performing the portable pool safety behaviours. Thus, it is important for water safety advocates to minimise the level of ambivalence in parents by presenting advantages or positives of supervision as outweighing the disadvantages or negatives. However, as identified in the current study, and previous research (Denehy et al., 2017), strategies are needed for those parents who struggle to know where to direct their attention when supervising multiple children. This may be overcome by encouraging parents to seek help from others when supervising around the portable pool, thereby ensuring an adult supervisor per child.

Behavioural beliefs unique to the disadvantages for fencing a portable pool were largely associated with the cost of pool fencing, followed by other disadvantages relating to; making a portable pool a permanent fixture and therefore limiting its use; extra hassles associated with fencing (i.e. council permits, inspections); not having the ability in rental properties to erect a pool fence; and not having the space to erect a fence. Likewise, unique behavioural beliefs describing the disadvantages pertaining to emptying and storing the portable pool were associated with the time involved in this process of draining the pool, drying and packing it down to only repeat the process again. Other disadvantages that followed were the excess water use and costs associated with that process and the size and design of the pool (i.e. the bigger the pool, the harder it is to empty and store). To counter parents’ beliefs of disadvantages pertaining to expenses and costs of pool fencing and emptying and storing of the pool after use, consumers should be educated on the true costs associated with the purchase of what may appear to be a low-cost option. Authorities could consider the development of a portable pool owner registry, as has been done in other areas of child product safety (Niven et al., 2023). Information compiled at point of sale, such as portable pool buyers’ contact details (i.e. email, phone number, home address), could be used to educate pool owners while also ensuring compliance with legal obligations of portable pool ownership.

### Approval and disapproval from different groups

Normative pressures were identified from both proximal, and distal individuals and/or groups. Family and friends were the most commonly identified individuals by parents as approving of all three portable pool safety behaviours followed closely by water safety advocates, councils, government, and health agencies. This is consistent with findings for parents’ supervision and restricting access to permanent pools (White et al., 2018). These normative beliefs indicate that not only are outcomes of performing portable pool safety behaviours imperative to contemplate, but the social pressure from significant others is an area that might be important to target, to encourage behaviours that may help reduce portable pool drownings.

Disapproval of others was also discussed, with the majority of parents being unable to think of anyone who would disapprove of performing the key target safety behaviours. In general, there was a clear normative expectancy that ‘everyone’ would approve of performing the key portable pool safety behaviours; that it is the ‘correct choice’ for the safety of their child, which is meaningful because violating your parental responsibility would result in social consequences (i.e. judgement; Bendor and Swistak, 2001), but also the potential negative impacts of a drowning incident. Additionally, given the importance placed on being perceived as a ‘good parent’ by others, positive peer pressure may be used as a catalyst for positive behaviour change (Waterkeyn and Waterkeyn, 2013), such as through the creation of community water safety champions.

Unique normative beliefs identified as disapproving, related to fencing, and emptying and storing portable pools after use. The disapproving beliefs were associated with landlords (fencing), and water authorities and drought affected people (emptying and storing). These perceived disapproving pressures were held by strangers, making these disapproving pressures more distal (e.g. water authorities) in contrast to the proximal sources (e.g. partners), as have been seen in other areas of childhood injury prevention (Quine et al., 2001; Scott-Parker et al., 2012). Given perceived social pressures from both close (family) and distal (lifesavers) individuals and groups, these should be considered in the context of portable pool safety behaviours by incorporating such normative belief systems into portable pool safety campaigns.

### Control beliefs: Preparation, avoiding distractions, and pool fencing guidelines

Finally, control beliefs surrounding the concept of planning as facilitating performing portable pool safety behaviours were identified. Parents were able to clearly describe those things that facilitated portable pool safety behaviours, particularly noting, for supervision within arm’s reach, being set up in a comfortable location, prepared, and having no distractions. Congruent with the identified facilitators, the main barriers to supervision within arm’s reach were distractions; lack of preparation; and not having the space, size, and location for the pool. In addition, some parents identified child factors as potential barriers to supervising, with one parent asserting ‘the sheer nature of them being toddlers is what makes it difficult, you know they can go quickly and quietly sometimes before you’ve even realised’. For some parents, it was the ensuring that you make a dedicated time for supervising that makes it easier, but also having another adult to share the supervising responsibility that facilitated close supervision. These identified themes can potentially be considered as elements of planning behaviour (Hagger et al., 2016; Norman and Conner, 2005). As such, overcoming barriers to supervision by creating a daily schedule which incorporates a dedicated time for swimming along with other commitments may help in minimising distractions that could interfere with a parents’ supervising duties.

For fencing the pool, the most common facilitators reported were having clear guidelines, awareness, and education surrounding what constitutes adequate portable pool fencing as well as the legal responsibilities to fence, which can vary between jurisdictions (Franklin and Peden, 2017). Government agencies and initiatives, therefore, need to ensure clear guidelines for portable pool owners to facilitate fence erection in accordance with safety regulations and requirements. Furthermore, cost-effective options or subsidies from the government were also reported as facilitators of fencing a portable pool. Some parents suggested that creating a ‘package deal’ where you purchase a pool with a fence included would facilitate the behaviour, eliminating the need for parents to do extra work to ensure their pool is safe, although fencing was seen to make a portable pool permanent, thus defeating its intended purpose.

### Barriers: Time, pool size, and pool construction

Unique to portable pools, the most commonly described barrier for emptying and storing was the time-consuming component, with parents likening it to just another thing that they have to do in already busy schedules. This finding was dependent upon the size and construction of the portable pool, with owners of smaller, lightweight pools less likely to identify any barriers associated with emptying their portable pool. Again, consumer education around portable pools may seek to highlight the differences in longer-term maintenance and safety requirements associated with pools of different construction.

### Strengths and limitations

The current study has several strengths including focusing on an at-risk group for drowning (i.e. children aged under 5) and examining, using a TPB belief-based framework, three key portable pool safety behaviours that have not been previously investigated qualitatively. Despite study strengths, several limitations should be noted. Discussions may have been susceptible to social desirability bias due to the nature of the topic, although the interviewer adopted a curious and nonjudgemental approach to promote an open and honest dialogue and avoid potential over-reporting of desirable responses (Davis et al., 2010). While purposive sampling was undertaken to ensure participants were recruited from diverse locations across Australia, recruitment was via convenience methods with some biases in those who were recruited for the study; predominately mothers of Caucasian decent and who were partnered, educated, employed, and residing in Metropolitan and suburban areas of Queensland, Australia. This may limit understandings of beliefs held by parents from different cultural groups, family structures, rural/remote settings, or lower socio-economic backgrounds, all of which have been shown to experience increased drowning risk (Peden et al., 2020, 2021). Utilising a panel company to create a representative sample may be a worthy consideration for future research on this topic.

## Conclusion

Research investigating portable pool safety behaviours is in its infancy and current findings fill a knowledge gap in the existing literature, providing evidence for salient beliefs which can be targeted to test whether manipulating the beliefs leads to positive changes in parents’ decisions towards portable pool safety behaviours. This information can be drawn upon when designing behaviour change messages and safety initiatives. Portable pool drownings are preventable and making them safer may ultimately save children’s lives.

## Supplemental Material

sj-docx-1-hpq-10.1177_13591053241275588 – Supplemental material for A qualitative analysis of parents’ beliefs about portable pool safety behaviours Supplemental material, sj-docx-1-hpq-10.1177_13591053241275588 for A qualitative analysis of parents’ beliefs about portable pool safety behaviours by Kyra Hamilton, Kim Dunn, Jacob J Keech and Amy E Peden in Journal of Health Psychology
