# Supplementary material for: A qualitative analysis of parents’ beliefs about portable pool safety behaviours
Source: J Health Psychol. 2024 Sep 18;30(9):2103–17. doi: 10.1177/13591053241275588 (PMC12322328; doi:10.1177/13591053241275588)
Supplement: sj-docx-1-hpq-10.1177_13591053241275588 – Supplemental material for A qualitative analysis of parents’ beliefs about portable pool safety behaviours [file sj-docx-1-hpq-10.1177_13591053241275588.docx]

Supplementary File 1: Consolidated criteria for reporting qualitative studies (COREQ): 32-item checklist

**Domain 1: Research team and reflexivity**

*Personal Characteristics*

|  |  | Page |
| --- | --- | --- |
| 1. Interviewer/facilitator | Which author/s conducted the interview or focus group? | 7 |
| 2. Credentials | What were the researcher’s credentials? E.g. PhD, MD | 7 |
| 3. Occupation | What was their occupation at the time of the study? | 7 |
| 4. Gender | Was the researcher male or female? | 7 |
| 5. Experience and training | What experience or training did the researcher have? | 7 |

*Relationship with Participants*

|  |  | Page |
| --- | --- | --- |
| 6. Relationship established | Was a relationship established prior to study commencement? | 7 |
| 7. Participant knowledge of the interviewer | What did the participants know about the researcher? e.g. personal goals, reasons for doing the research | 7 |
| 8. Interviewer characteristics | What characteristics were reported about the interviewer/facilitator? e.g. Bias, assumptions, reasons and interests in the research topic | 7 |

**Domain 2: study design**

*Theoretical framework*

|  |  | Page |
| --- | --- | --- |
| 9. Methodological orientation and Theory | What methodological orientation was stated to underpin the study? e.g. grounded theory,  discourse analysis, ethnography, phenomenology, content analysis | 5, 8 |

*Participant selection*

|  |  | Page |
| --- | --- | --- |
| 10. Sampling | How were participants selected? e.g. purposive, convenience, consecutive, snowball | 5-6 |
| 11. Method of approach | How were participants approached? e.g. face-to-face, telephone, mail, email | 6 |
| 12. Sample size | How many participants were in the study? | 6 |
| 13. Non-participation | How many people refused to participate or dropped out? Reasons? | 5,6 |

*Setting*

|  |  | Page |
| --- | --- | --- |
| 14. Setting of data collection | Where was the data collected? e.g. home, clinic, workplace | 7 |
| 15. Presence of non-participants | Was anyone else present besides the participants and researchers? | 6,7 |
| 16. Description of sample | What are the important characteristics of the sample? e.g. demographic data, date | 6 |

*Data collection*

|  |  | Page |
| --- | --- | --- |
| 17. Interview guide | Were questions, prompts, guides provided by the authors? Was it pilot tested? | 7,8, supplementary file 2 |
| 18. Repeat interviews | Were repeat interviews carried out? If yes, how many? | 8 |
| 19. Audio/visual recording | Did the research use audio or visual recording to collect the data? | 7 |
| 20. Field notes | Were field notes made during and/or after the interview or focus group? | 8 |
| 21. Duration | What was the duration of the interviews or focus group? | 7 |
| 22. Data saturation | Was data saturation discussed? | 8-9 |
| 23. Transcripts returned | Were transcripts returned to participants for comment and/or correction? | 8 |

**Domain 3: analysis and findings**

*Data analysis*

|  |  | Page |
| --- | --- | --- |
| 24. Number of data coders | How many data coders coded the data? | 7 |
| 25. Description of the coding tree | Did authors provide a description of the coding tree? | 8 |
| 26. Derivation of themes | Were themes identified in advance or derived from the data? | 7 |
| 27. Software | What software, if applicable, was used to manage the data? | 7 |
| 28. Participant checking | Did participants provide feedback on the findings? | 8 |

*Reporting*

|  |  | Page |
| --- | --- | --- |
| 29. Quotations presented | Were participant quotations presented to illustrate the themes / findings? Was each  quotation identified? e.g. participant number | 9-19, supplementary file 3 |
| 30. Data and findings consistent | Was there consistency between the data presented and the findings? | 19-24 |
| 31. Clarity of major themes | Were major themes clearly presented in the findings? | 9-19 |
| 32. Clarity of minor themes | Is there a description of diverse cases or discussion of minor themes? | 9-19 |

Developed from: Tong A, Sainsbury P, Craig J. (2007). Consolidated criteria for reporting qualitative research (COREQ): a 32-item checklist for interviews and focus groups. *International Journal for Quality in Health Care, 19(6)*, p. 352

Supplementary File 2: Questionnaire and Interview Protocol

**Interview# ____________**

**Time ______________**

**Date/Day ______________**

**Location ______________**

**DEMOGRAPHIC INFORMATION**

The following personal details will not be used for identification purposes, but will help describe the sample of participants

1. Gender:
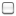
 Male
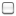
 Female
2. Age: __________years
3. Postcode of Residence: __________
4. Marital Status:

| Never married | Married registered | Married defacto | Separated/Divorced | Widowed |
| --- | --- | --- | --- | --- |

1. How many children 5 and under do you have? _________________________________
2. What is the age and gender of each of your children? ________________________
3. Employment Status: (Please tick as many that apply to you)

| Unemployed/Home duties | Casual/Part time work | Full time work | Part time student | Full time student |
| --- | --- | --- | --- | --- |

1. Highest level of education achieved:

| Completed junior school (yr 10) or less | Completed senior school (yr 12) | TAFE/Diploma | Undergraduate University degree | Postgraduate University degree |
| --- | --- | --- | --- | --- |

1. Family taxable income range (please ✓ a box):

❑ Nil – $18,200 ❑ $18,201 - $37,000 ❑ $37,001 – $80,000

❑ $80,001 – $180,000 ❑ >$180,001

1. Do you come from a Non-English Speaking Background?
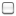
Yes
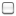
 No

| If yes, what is your background: |
| --- |
|  |

1. Occupation?____________________________________________________________________

Interview Questions

| **Supervising young children around the water** | |
| --- | --- |
| ***Behavioural Beliefs - Attitude*** | |
| Number | Question |
| 1 | What do you see as the ***advantages*** of supervising your young child within arm’s reach around your portable pool? |
| 2 | What do you see as the ***disadvantages*** of supervising your young child within arm’s reach around your portable pool? |
| 3 | What else comes to mind when you think about supervising your young child within arm’s reach around your portable pool |
| ***Normative Beliefs - Perceived Norm*** | |
| 4 | Who are the individuals or groups of people who would ***approve*** of you supervising your young child within arm’s reach around your portable pool? |
| 5 | Who are the individuals or groups of people who would ***disapprove*** of you supervising your young child within arm’s reach around your portable pool? |
| 6 | Are there ***any other individuals or groups that come to mind*** when you think about supervising your young child within arm’s reach around your portable pool? |
| ***Control Beliefs - Behavioural Control*** | |
| 7 | What are the factors that ***make it easier*** for you to supervise your young child within arm’s reach around your portable pool. |
| 8 | What are the factors that ***make it harder or more difficult*** for you to supervise your young child within arm’s reach around your portable pool. |
| 9 | Are there any other issues that come to mind when you think about supervising your young child within arm’s reach around your portable pool? |
| **Emptying and storing portable pools safely when they are not in use** | |
| ***Behavioural Beliefs - Attitude*** | |
| 1 | What do you identify as the ***advantages*** emptying and storing your portable pool safely when not in use? |
| 2 | What do you identify as the ***disadvantages*** of emptying and storing your portable pool safely when not in use? |
| 3 | Is there anything else you associate with emptying and storing your portable pool safely when not in use? |
| ***Normative Beliefs - Perceived Norm*** | |
| 4 | Who are the individuals or groups that would ***approve or want you*** emptying and storing your portable pool safely when not in use? |
| 5 | Who are the individuals or groups that would ***disapprove*** of you emptying and storing your portable pool safely when not in use? |
| 6 | Are there any other individuals or groups that come to mind when you think about emptying and storing your portable pool safely when not in use? |
| ***Control Beliefs - Behavioural Control*** | |
| 7 | What are the factors that ***make it easier*** for you to empty and store your portable pool safely when not in use? |
| 8 | What are the factors that ***make it difficult*** for you to empty and store your portable pool safely when not in use? |
| 9 | Are there any other issues that come to mind when you think about emptying and storing your portable pool safely when not in use? |
| **Ensuring adequate fencing is in place to restrict access to portable pools deeper than 30cm** | |
| ***Behavioural Beliefs - Attitudes*** | |
| 1 | What do you identify as the ***advantages*** ensuring adequate fencing is in place to restrict access to portable pools deeper than 30cm? |
| 2 | What do you identify as the ***disadvantages*** of ensuring adequate fencing is in place to restrict access to portable pools deeper than 30cm? |
| 3 | Is there anything else you associate with ensuring adequate fencing is in place to restrict access to portable pools deeper than 30cm? |
| ***Normative Beliefs - Perceived Norm*** | |
| 4 | Who are the individuals or groups that would ***approve or want you*** to ensure adequate fencing is in place to restrict access to portable pools deeper than 30cm? |
| 5 | Who are the individuals or groups that would ***disapprove*** of you ensuring adequate fencing is in place to restrict access to portable pools deeper than 30cm? |
| 6 | Are there any other individuals or groups that come to mind when you think about ensuring adequate fencing is in place to restrict access to portable pools deeper than 30cm? |
| ***Control Beliefs - Behavioural Control*** | |
| 7 | What are the factors that ***make it easier*** for you to ensure adequate fencing is in place to restrict access to portable pools deeper than 30cm? |
| 8 | What are the factors that ***make it difficult*** for you ensure adequate fencing is in place to restrict access to portable pools deeper than 30cm? |
| 9 | Are there any other issues that come to mind when you think ensuring adequate fencing is in place to restrict access to portable pools deeper than 30cm? |

Supplementary File 3: Summary of Concept, Key Themes, and Supporting Quotes of Behavioural, Normative, and Control Beliefs for the three target portable pool safety behaviours

Table 3.1

*Summary of Concept, Key Themes, and Supporting Quotes of Behavioural, Normative, and Control Beliefs for Supervising Within Arm’s Reach*

| Concept | Key themes | Supporting quotes |
| --- | --- | --- |
| **Behavioural Beliefs** |  |  |
| Advantages |  |  |
|  | Quick response to prevent incident or injury | “For me it’s being able to respond quickly, that’s the main advantage is being able to quickly respond and for me that goes a little bit further in the sense that I really don’t want my children to be traumatised by water; I try to keep water a really positive thing for them. We water-ski on the river so we’re pretty water people in summer, so I really try to make sure that any experience they have around water at home that’s in the portable pool is really positive. So, the quicker I can respond to something that might go wrong is going to help me achieve that.” *– P03.*  “Definitely the advantages is that kids tend to just slip, I’ve seen it numerous times even with my own children and they just go under so it’s not something, like they don’t sort of yell out “I’m going to fall over” and not make a sound about it. So, they tend to just slip and fall under in water and they do it very quickly so it’s just one of those things. Or they fall backwards whether that be assisted sometimes by other children in the pool, so yeah they tend to just especially when I think the two year old range they slip or fall backwards very quickly so that’s where I think it’s very important to stay in arms distance.” *– P08.*  “um if anything was to happen you would be able to, you know if they were to dip under the water or anything you will be able to grab them quickly enough to prevent them I suppose from swallowing any water or anything further happening.” – *P11.*  “The advantages would be that if something does happen and they sort of go under water you can grab them straight away, so they are less likely to have an emersion injury or something like that.” - *P14.*  “To me its personal, it’s the safety of my child, I can’t think of anything worse than burying my own child due to my own negligence.” – *P15.* |
|  | Engagement and bonding | “I think there’s obviously the engagement; so actually, being present and being part of their activities” – *P01*.  “I mean it’s a good sort of social, interactive type experience that is good for family and friends to bond and relax and all that sort of stuff.” – *P06*.  “I’m also a yoga teacher it’s a matter of mindfulness and that when you are engaging with a young child in an activity like that you are being in the moment and being with them and engaged rather than being distracted um, so really enjoying being involved in that activity which then increases the safety side of it cause you are more aware of the dangers and closer proximity.” *–* *P12*.  “you can be sort of part of the play and be enjoying the time with them as well, so it’s like a good bonding experience” – *P14.* |
|  | Educational | “you can reinforce safe behaviours and non-safe behaviours. So, if they’re climbing or jumping or being silly you know you can show them the right way to do things.” *– P01.*  “like in terms of like when we’re playing outside with my daughter in the water, I’ll often talk about maybe the temperature of the water or if we’ve got containers that we’re playing with as well in the pool, like about how full or how empty they are. Whereas obviously if you’re on the other side of the yard from the child while doing that it’s harder to do that, it’s not like you can really have a conversation with them. You can’t have a conversation with them about if they’re doing something silly in the pool, about being safe in the pool, that sort of thing.” *– P02.*  “Making sure they are not getting into trouble, teaching them kicking, and paddling and blowing bubbles and those sorts of things, so you can sort of teach them water safety concepts.” *– P14.* |
|  | Child’s sense of security | “the advantage is that you offer them security so they’re in an environment that they’re unsure of as well and so by being very close at hand if they wish to exit the pool quite often quite young ones need assistance exiting the pool, so you can do so immediately. Or if they’re just unsure they can just exit and enter the pool a lot when they’re testing out the new environment and that can be a source of danger as well when they exit and enter the pool.” *– P08.*  “The child feels safe cause they know that mums right there.” *– P10.*  “them knowing and feeling safe cause they know your there.” *– P14.* |
| Disadvantages |  |  |
|  | Can’t do anything else while supervising and it can be a boring task | “I guess honestly this is like the rude part about being a parent but it’s just sometimes you just don’t want to. Like you don’t want to, especially in the Territory if you’re outside chances are it’s going to be hot and especially if it’s in the sun, like if there’s no shade over you while you’re sitting there watching, but we set ours up so that’s not really an issue for us. I guess general just because a kid wants to do something doesn’t always mean a parent wants to and so it’s obviously just the hassle factor for the parent I reckon.” *– P02.*  “Nothing else gets achieved, it literally is just sitting there and watching them and there’s always things that need to be done. I know it sounds really terrible but it’s true there’s lots that needs to be done and sometimes it’s not exactly what I really would like to be doing or feel like I should be doing to just sit and watch.*” – P03.*  “I suppose that the only disadvantage is that’s all you can do, you can’t really be doing anything else apart from that one job.” *– P11.* |
|  | Creates a lack of independence and confidence | “And you know they have to learn a little bit of independence and responsibility as well; knowing what is right and what is wrong so I think there is a disadvantage in that for someone that’s around that four or five age group that has met their developmental milestones and is appropriately acting as a four or five year old.” *– P01*  “Disadvantage is that your child might become reliant on you if you always prevent them from going face down. But I think that just comes with as they get a little older you see their capabilities getting a little better kind of let them try to prevent themselves from going face down in the water.*”- P07.*  “Disadvantages, probably not encouraging there confidence, hovering a little bit too much, um when they are trying to learn to swim sometimes you gotta, you know, you do have to let them have some space and get them confident in their own safety in the water. Yeah that would be the only disadvantage.” *– P13* |
|  | Having other children to look after | “Well I guess me being a busy mum it’s that I can’t do anything else but watch them and for me I then have to decide what I do with the younger baby. Do I sit the younger baby with me and therefore I might not be able to help her as quickly or does that mean I have to have the baby a little bit further away from me? I guess that’s the disadvantages with a second child.” *– P09.* |
|  |  |  |
| **Normative Beliefs** |  |  |
| Approve |  |  |
|  | Water safety advocates | “I think places like um swimming lessons and you’ve got Laurie Lawrence, his does that song about the 5 stay alive thing, umm so those sort of things sort of trying to ingrain those behaviours and so those kind of organisations would be pleased by the within arm’s reach thing..” *– P10*  “lifesavers are big advocates for water safety, so I probably think that they’re the two groups that certainly would be big advocates for supervision within an arm’s reach.” *– P01.* |
|  | Family | “family would expect that I would be supervising them adequately. My husband would certainly expect that that is what I was doing while he’s not here.” *– P03.*  “I feel like most of our immediate family would approve of it just because we grew up around with our own personal pool and so like we’re all fine with being in pools, being supervised hopefully as long as supervised we’re all cool with it. And so I think everyone in my immediate family would approve of it.” *–P07.*  “Okay well definitely most of our immediate family would definitely do the same thing, like I would leave them in their care comfortable that they would do the same thing.” *– P08.* |
|  | Friends | “A lot of my friends who have young children would also I think be quite strong on that side of things as well.” *– P02.*  “Yeah almost all my friends’ group would say the same thing definitely.” *– P08*  “I suppose the social circle that I am in is, we are all mums, you know adoring mums so we would do anything to keep that little one safe.” – *P12.* |
|  | Councils, government and health agencies | “Department of family services, and all those places who are very strong on that.” *– P10.*  “I would say the council and the government would approve of yeah supervising your children.” *– P11*.  “health workers, people perhaps who have seen the consequences of children who have not been supervised and there’s an adverse outcome.” *– P14.* |
|  | Other parents | “I guess a lot of other mums would want that to happen if their child was with you.” *– P09.*  “Other parents of young children and grandparents would approve of being close and supervising.” *– P14.* |
|  |  |  |
| Disapprove |  |  |
|  | No one would disapprove | “yeah, no one because I wouldn’t have them in my social circle, yeah no.” *– P12.*  “I can’t think of anyone who would be disapproving of me doing that. Honestly I can’t imagine anybody who would think it was a bad idea.” *– P03.*  “No one within my circle.” *– P04.* |
|  | Older generations | “I just think my definition of supervising is very different to I know their grandmother’s definition. I think that they’re a little more open to, I wouldn’t say risk taking, it’s probably not the right word but they’re probably a lot more tolerant of different things than what I am I suppose. So I know that I’m certainly a lot more water-conscious around and even just at the beach than what they were.” *– P01.*  “generational relatives like grandparents or something that might think you’re mollycoddling your children and just letting them sink or swim I guess, if that’s the old fashioned attitude they might have perhaps, that’s the only negative think I can think of anybody saying anything like that.” *– P06.*  “Socially, I guess you’d be labelled as a helicopter parent, um but I can’t think of anything else that would be classed as a disapproval. Yeah really got that social stigma of helicopter parent, you can’t leave your kid, and should you be able to move more than arms reach away from them type thing, like to play into that stereo type so to speak.” *– P10.* |
|  |  |  |
| **Control Beliefs** |  |  |
| Facilitators |  |  |
|  | Comfortable location | “Well ventilated, it’s large enough for you to be able to sit around the pool. It’s comfortable, maybe it’s a shaded area or whatever the case may be so that you’re not having to… you know if you feel comfortable, you’re more likely to actually stay there and stick around for a bit longer” *– P01.*  “Well I think it’s important to obviously be smart about how you set up your portable pool so the location of it, whether it’s under shade or whether it’s in a, depending on what area like whether there’s seating nearby for you, how easy it is for you like if you’ve got a portable chair or something that you can take over and sit within their general vicinity.” – *P02*.  “Making sure that you’re comfortable i.e. you’re in a shady spot, nice chair, you’re out of splashing distance.” *– P04*. |
|  | Being prepared and organised | “Obviously being well set up like if you’ve got, like if you’re worried about expecting a phone call making sure you’ve got the phone right there next to you. Having a bottle of water there as well in case you get thirsty so that you don’t have to leave, basically having what you need there. I think that for me in our situation with a pool size we’ve got I think that really comes down to us as parents just making sure you’re ready for that, making sure you’re prepared.” *– P02.*  “I try and make sure I’ve got everything with me that won’t distract me, and I leave her.” *– P09.*  “I suppose if I’ve got everything out there, like our hat and our towels and all those kinds of things so I’m not thinking oh shot I need to run off and grab xyz.” *– P12.*  “If you’ve got everything you need out there, so things like you know snacks, water, sunscreen, nappies in case you need them, so things like that, if you got things that are accessible that you might need, it will be easier to just sit and stay in the one spot and not have to, you know towels all that sort of thing.” *– P14.* |
|  | Having no distractions | “Reducing distractions around the area and possible interferences.”- *P05.*  “Just don’t take the phone outside really, just go out there and interact really is the best thing to do, just play with them. Either be in the pool or beside the pool and our daughters at that really playful age so we’re always constantly engaging, probably too much if you asked her.” *– P06.* |
|  | Dedicating time | “Actually, dedicating the time for the activity not scheduling or trying to do too many things at once, it certainly makes it easier for supervision.” *– P01.*  “Setting aside specific times to play in the pool.” *– P05.* |
|  | Having another adult to help | “Probably having other people around as well, so other adults so that you know if you do need to go to the bathroom or go and grab something or you know go and do some jobs or something then there can be someone there as well, it will probably be easier and its more enjoyable having another adult to talk to as well.” *– P14.*  “You know two adults is better than one so that’s always a good thing. Two sets of eyes is better than one and that kind of thing especially if it’s more than, for us it’s just been us and our daughter so it’s been two adults and one child but certainly if there’s a few involved it would be good to have at least two adults with eyes on the situation.” *– P06.* |
| Barriers |  |  |
|  | Distractions | “I guess that’s the difficulty because I guess it’s all very well to say have the phone there so that you can answer it if you need to but at the same time if it’s… Like I know that I get sucked in sometimes to social media or looking at things on Pinterest and not paying attention necessarily to exactly what’s going on around me. And because as it is if you are … Especially if sitting by the pool is not exactly what you want to be doing then it’s all too easy to sit there and want to be doing something else and therefore doing something else while you’re out there. I think even sometimes I get talking on the phone or looking at things on your phone that can take your mind away from where it should really be which is probably on the kids while they’re in the pool.” *– P02.*  “First thing would be distractions, so in a domestic situation you’ve got potentially dogs running around barking, making noises, distractions with mobile phones is probably a big one. So yeah really distraction is probably, would be the number one thing or even just complacency and the whole routine, that’s sort of how it goes.” – *P06.*  “I think the majority of the time the phone is distracting in any capacity. I think the least is probably a phone call because then I can still see her, but I probably won’t be able to predict her actions as well when I’m distracted on the phone. But definitely text and all that you’re not looking at your child, you’re looking at your phones.” *– P09.* |
|  | Not being prepared or organised | “Not planning, you know having to go off and get the washing off the line, or I’ve just got to put potatoes on or whatever the case may be for dinner and those types of things certainly make it very difficult.” *– P01.*  “Probably if you don’t have all your stuff, sorry, organized, if you have to be running away getting things all the time or you know you’re not prepared.” *– P14.* |
|  | Child factors | “young child in that younger age they’re still quite impulsive and you can’t really rationalise with them.” *– P01.*  “I think the main thing that I find that makes it difficult is when they first get in, as I said there’s no, sometimes there’s no warning to it. Sometimes they’ll just run out and they’ll get in fully clothed, like sometimes there could be no warning, that’s the hardest thing that I find is that sometimes they’ll just want to go. If it was more like an in-ground pool with an actual fence around it and it was in the same spot all the time, that gets eliminated a bit more, they’re crafty but it does reduce it a little bit. So, the sheer nature of them being toddlers is what makes it difficult, you know they can go quickly and quietly sometimes before you’ve even realised.” *– P03.* |
|  | Having the space, size and location | “Yeah so enough space for both the pool, the child and the parent to occupy.” *– P05.*  “Well I guess having the space to have the pool and to be able to sit around it.” *– P09.*  “Obviously the location in the pool, umm I wouldn’t have thought it’s particularly a hard thing to do apart from obviously the size and the location of where it is put as to why it would be difficult.” *– P10.* |

Table 3.2

*Summary of Concept, Key Themes, and Supporting Quotes of Behavioural, Normative, and Control Beliefs for Fencing Portable pools Deeper Than 30cm*

| Concept | Key themes | Supporting quotes |  |
| --- | --- | --- | --- |
| **Behavioural Beliefs** |  |  |  |
| Advantages |  |  |  |
|  | Prevention of drowning and safety | “Obviously the safety of the child, knowing that they can be out in the backyard, knowing that they can be in that environment and still be physically separated I suppose from something that’s potentially dangerous for them.” *– P01.*  “The advantages would be to prevent drowning I guess, that’s kind of the point. To stop kids unknowingly from entering the pool I guess that’s the biggest thing is that they may unknowingly enter the pool and yeah prevent them from drowning really.” *– P08.*  “The biggest advantage is the fact that you are negating the majority of the possibilities of someone drowning in your pool.” *– P10.* |  |
|  | Peace of mind | “Obviously it’d make it easier to walk away from the backyard area if you had a child out there unsupervised if you had to for a short period of time, because if you know a fence is up and you know a fence is stable you’re not going to worry about walking away. It’s going to make it easier to you know if they’re in the backyard to walk inside to grab the phone and walk back out, like it’s not, that won’t be an issue.” *–* *P02*.  “Peace of mind.” *–* *P05*.  “That peace of mind if you do have to take them out and duck off to do something that they can’t just make their way back without out you being aware that they are attempting to. Peace of mind.” – *P10*. |  |
| Disadvantages | Expensive and costly | “I think when you actually look at it the fencing themselves is actually more expensive than the pools in most circumstances so you can buy, you know you can pick them up for twenty dollars, thirty dollars at the shops for these large, up to a metre depth type pools and the fencing for it is outrageously expensive per panel.” *– P01.*    “The disadvantage would be the cost of fencing it.” *– P09.*    “It’s expensive if you have to and then you have that issue where what do you do, do you have the pool and run the risk, or put a proper pool in cause nobody wants to pay for a fence for a pool that can fall apart in 5 minutes.” *– P10.* |  |
|  |  |  |  |
|  | Becomes a permanent fixture | “portable pool you can move its location so it doesn’t kill your grass, so you can get a different area now and then, you can move it depending on the time of the day, you know the shade in this area versus the shade in another area. But if you’re putting a fence there you’re really limiting your purpose of the portable pool, it doesn’t become a portable pool anymore.” *– P10.*  “making it a permanent structure.” *– P13.*  “It would mean you have to keep the pool in the one spot, which defeats the purpose of it being portable I guess.” *– P14.* |  |
|  | Creates additional hassles | “You then have to depending on where you’re putting your fence there could be permits, processes that you need to go through with your council as well. Then there’s inspection, so my understanding is that you have to have pool fencing, if it’s used for the actual purposes of pool fencing, inspected and those types of things as well.” *– P01.*  “Sometimes things like when you’re trying to take numerous things out to the pool area umm, plus lugging a child you don’t have enough hands then trying to unlatch the gate in order to get in, which I know my husband as told me off a few times cause I have propped the gate open to move to and fro and I don’t know if his telling the truth but his told me that the council have drowns that fly around and check peoples pool areas a secure, not sure if that’s true or not but that’s what his told me umm so that scared me a bit but yes I know that can be quite a pain when I’m trying to go you know if we are having a BBQ or something in the pool area and I’m trying to go backwards and forwards, backwards and forwards, that can be annoying.*” – P12.* |  |
| **Normative Beliefs** |  |  |  |
|  |  |  |  |
| Approve |  |  |  |
|  | Council, government and health agencies | “You know councils approve of it I think it’s well, unfortunately I’m a bit cynical, I think it’s more activity for them, I think construction companies, I think anyone who can actually make some money out of it I think would approve of fencing type things and guidelines being put in place.” *– P01.*  “I suppose advocacy groups that might have started due to the fact that they’ve experience someone drowning near to them or lost someone close to them, they would probably advocate that you do it.”  *– P08.*  “All levels of government, local government especially cause they are the ones who watch it pretty strictly.” *– P10.* |  |
|  | Family | “And of course, family would definitely approve of adequate safety, they don’t want things to happen to the family by accident.” *– P07.*  “Family has an expectation that if it’s too big they don’t want to put their own kids at risk so they would expect you to have it blocked of in some way, fenced in some way.” *– P10.*  “I can’t imagine that people would really disapprove … I mean not when it comes down to your safety” *–P01.* |  |
|  | Friends | “Certainly, where it stands at the moment it would be mainly our friends.” *– P02.*  “everyone we socialise with.” *– P12.*  “Close friends.” – *P14.* |  |
|  | Majority of people | “I think obviously legislatively and I guess probably the wider community here it would make more sense if the pool was in our front yard or in a more open space where it’s accessible for other people in the community then obviously that’d be more important to them.” *– P02.*  “I feel like the majority of people in general would approve of that especially I could see like anyone that would, could potentially be found liable if something were to happen would definitely approve of fenced in areas. Whether it be a whole apartment complex board or something or community something, just anyone that could be found liable if something were to happen. They would want to make sure that no safety measures are in place that they don’t, they’re not taking the hit for something.” *– P07*. |  |
|  | Neighbours | “I would say an entire neighbourhood would be thankful, as I said before it’s not just my own kids that I have to worry about like if somebody happened to get in to your yard, which is totally reasonable to happen, the liability and things I would say that anyone in the community would be happy that it was fenced adequately.” *– P07*.  “Guess people who may have access to your property, like your neighbours but we are fenced all around our property anyway.” *– P14*. |  |
|  | Water safety advocates | “I think you’re going to have all of your advocating groups, your Laurie Lawrence’s, your lifeguards” *– P01*. |  |
| Disapprove |  |  |  |
|  | Landlords | “I’m thinking from the landlord’s perspective, a very cold-hearted one who doesn’t want the, you know like they have a set standard for their property and maybe a fence could I don’t know tarnish the image or the marketing or what they want.” *– P05.*  “I think probably it would be well possibly the rental companies that we deal with; they probably may not be too impressed with that.” *– P02.* |  |
|  | No one would disapprove | “I couldn’t see anyone disapproving of that.” *– P07.* |  |
| **Control Beliefs** |  |  |  |
| Facilitators |  |  |  |
|  | Clear guidelines, awareness and education | “Well first and foremost clear you know “this is your legal obligations”, like clear information as to what is expected and what the requirements are. And perhaps that information exists, I don’t know. Yeah clear knowledge of what was required. It’s hard to sort of say what else would make it easier without knowing what it would have to be.” – *P03*.  “I think having some very set legislation around it umm I think makes it easier for people because there is very strict guidelines around what is required, I know that they come around and do a check, it must be when you sell the house, to make sure you got that pool safety certificate, which is very helpful.” *– P12.*  “a little bit more education I think, I don’t think we actually really aware, we all know that if we get an in ground pool we have to have a fence but we don’t know, I don’t think it’s as widely accepted or known that if you buy a little portable pool from BCF or something you have to get a fence, so yeah.” *– P13*.  “more campaigns about it so it increases the awareness and so you think about it a bit more for some of those smaller pools.” *– P14.* |  |
|  | Make cost effective or subsidise | “making them more cost effective, that certainly makes it … You know finding a way to make it more cost effective for portable pools if that’s the way that it’s going to go then that’s where it needs to… You know cost is a huge thing.” *– P01.*  “Well I think if you were, I think the cost or some kind of subsidy for putting a fence around your pool.” *– P14.*  “Cost is a concern; you know is it going to cost me a thousand dollars to put a fence around my forty-dollar blow up pool? The cost versus the outcome, or benefit versus you know whatever that relationship is, like is that worth it? So, for a forty-dollar blow up pool that the kids can splash in it, I’m going to have to put a thousand bucks worth of fence around it then no, that’s a no from me.” *– P03.* |  |
|  | Package deal, pool and fence sold together | “Maybe a fencing pack along with the pool. Like all jokes aside if you could literally buy a cheaper version of a fencing pack that you could securely put up and easily put up along with the cheap and small pool then that would be fantastic because then it comes down to ease of use and cost in the end and something that was not so permanent because then people who are living in rental properties could access it as well.” *– P08.*  “It would be a package deal I guess, if you buy the pool the fence comes with it, so it’s not like you have to do separate work, the manufacturers of the pool, people promoting the pool have the whole entire package. That would be great.”- *P13.* |  |
| Barriers | Difficult and a hassle | “The practicality of it, so whether it’s buying and then taking the time to set it up yourself or paying for somebody else to organise that and then also whether it’s going to restrict your access to something else in your yard or your life in general, that would obviously make you less likely to do it. It’s not for us an issue but aesthetically as well, some people may be keen for a certain type or a certain design in their yard and it may, having fencing up may hinder that, but that’s not an issue for us.” *– P02.*  “I guess any potential issues with either your neighbours, having an issue with maybe a contractor that’s doing it, and also any resistance you might encounter trying to get approval. You might be trying to put a pool fence down on your property where you might have an easement or another restriction that you have to work around for approval.” *– P06.*    “I think it would create more of a hassle then yeah then getting then just having the pool by itself, we would be less likely to have a portable pool if we had to have a fence.” *– P11.* |  |
|  |  |  |  |
|  | Unclear requirements and lack of knowledge | “I guess a problem could be if someone thinks if they put a portable fence up and to them it could be, they think it’s adequate but to someone else it could seem like inadequate fencing. And so it’s just, I don't know if there's any guidelines for what actual fencing would consider for a portable pool and so people’s ideas of that could be different. And some people could have issues of other people’s fencing you could say.” – *P07.*  *“*Not knowing exactly what you are supposed to have, there is so many different types out there that you’re not quite sure what will and won’t actually be passed as adequate fencing.” *– P10.*    “not knowing what is required, that makes it difficult.” *– P02.* |  |
|  | Cost | “Cost obviously would be the big one, it can be pool fencing is not particularly cheap, and to do it properly, otherwise there is no point in doing it anyway, it’s not particularly a cheap exercise.” *– P10.*  “I think just more like if there was a financial burden to do so would be difficult.” *– P07.*    *“*the cost of the fencing and of installing the fencing.” *– P02.* |  |
|  | Rental property | “We’re a defence family so we constantly move, so obviously if we were to move, we’re due to move at the end of this year so when we move if we had fencing up in one property obviously making sure that that’s portable and that it doesn’t actually alter the state of the property we’re in otherwise we’ll be charged to rectify that” *– P02.*  “We rent and we’re about to have to move house, is it something I can take along with me? Like whose responsibility is it? It sort of becomes an infrastructure kind of thing and when you rent that can be quite difficult to navigate” *– P03.*  “A disadvantage too is the fact that a lot of people live in rental properties, so you don’t have permission to change or add things like permanent fencing or fencing structures to facilitate the requirements, so that’s definitely a disadvantage.” *– P08.* |  |
|  | Space to erect a fence | “the actual physical space.” *– P08.*  “find a spot in our backyard that we can adequately fence.” *– P09.* |  |
|  |  |  |  |
|  |  |  |  |

Table 3.3

*Summary of Concept, Key Themes, and Supporting Quotes of Behavioural, Normative, and Control Beliefs for Emptying and Storing Safely*

| Concept | Key themes | Supporting quotes |
| --- | --- | --- |
| **Behavioural Beliefs** |  |  |
| Advantages |  |  |
|  | Prevents drowning and ensures safety | “it’s taken away from the child, so the child couldn’t possibly go in the pool without your knowledge or fall in the pool without you knowing.” *– P11.*  “safer so my kid doesn’t stumble upon the body of water haphazardly.” *– P05.*  “The advantage is that if you do it straight away and you just make a habit of it, it’s done, you don’t have to think about it, no one’s going to come out and drown later, also I think sometimes it’s easy to get distracted with everyone running around and your trying to dry everyone, it can be easy to forget to do it cause there’s lots of things going on with young children in the house but yeah I think mainly the safety aspect is the best thing about storing it straight away and then you don’t need to think about it anymore.” *– P05.* |
|  | Prevents mould, algae, bacteria and maintains water hygiene | “well because it would just become mouldy you know cause it’s not been filtered and it doesn’t have the chemicals in it to keep it clean, so it would just become disgusting then of course my daughter would be attracted to playing with the mould in the water, and so yeah it’s the hygiene, hygiene would be a big factor. I don’t know if that green mould algae stuff is overly hygienic.” *–* *P12*.  “The fact that it doesn’t get feisty, diseased and disgusting.” – *P15*. |
|  | Prevents mosquitos | “Mosquitos certainly up here in Queensland, so it becomes an area for mosquitos to breed if it’s not emptied out.” *– P08.*    “No mosquitos to lay eggs and larvae and those bits and pieces.” *– P01.* |
|  | Preserves the life of the pool | “pool will last longer if you empty and store it properly cause it’s not sitting in the sun and it’s not being perished by the water and those sorts of things.” *– P10.* |
|  |  |  |
|  | Reuse the water to feed gardens and lawns | “we use the water to then water the grass and plants and those bits and pieces as well so it’s sort of we sort of see it as doing our bit, you know the kids get to play but then we’re recycling that water I suppose and feeding the plants at the same time which is something in Queensland where water is really precious for us and we don’t get much of it so yeah I think that’s probably one of the biggest advantages.” *– P01.*  “I guess probably just being more mindful of using that water for the actual garden and places where it’s needed, is a consideration.” *– P14.* |
| Disadvantages |  |  |
|  | Time and effort | “It’s a pain in the butt, because you got to empty it and then you got to wait for it to dry and then put it away, and then when you do want to use it then you have to go to the effort especially with a blow pool or one of those canvas ones of rebuilding it from scratch every time.” *– P10.*  “another disadvantage is that when you want to use it again you gotta go through the whole process of getting it back out and refilling it and all of that.” *– P11.*  “I think it’s just another task or job to do that takes time that you just feel like you’re so busy anyway that you think ah.” *– P14.* |
|  | Excess water use and cost | “Well I guess the disadvantage is that it’s not particularly water-safe to fill up a pool and empty it daily. Sometimes it could be two or three times a day and I guess it’s not water-wise and I guess that’s another job for me to do every time we finish playing I have to pour the water out and find somewhere to put it that’s safe.” *– P09.*  “Water use because obviously each time we fill it up and each time we empty it it’s using water so that’s a cost for water but then also environmentally how much extra water we’re using, whether it’s really the best option.” *– P02.* |
|  | The size and design of the pool | “Well one would be the size of the pool.” *– P02.*  “The other disadvantage is you’d have to with that particular thing it’s actually a pain to stand up, so it’s designed to be laid flat, you can turn it over but then the kids jump on it and break it. So it’s actually the way it’s designed to sit open and collect water is the best way for it to sit and so the disadvantage is the way the pool is built almost it doesn’t store very easily. It doesn’t stand upright so that’s definitely a disadvantage I suppose.” *– P08.* |
|  |  |  |
| **Normative Beliefs** |  |  |
| Approve |  |  |
|  | Family | “My family group would expect that if they came round and their children were in my house but there wasn’t something they had to worry about outside in the backyard that I was letting them unsupervised or not anywhere near water that was unsupervised or something like that.” *– P08.*  “My husband he would approve me of doing more often than I do.” *– P11.*  “Well I think all of our family members are supportive of us doing that straight away, just so it decreases that risk of someone drowning in there.” *– P14.* |
|  | Council, government and health agencies | “Council would approve of it as well from an insect control type bits and pieces.” *– P01.*  “family services those sorts of things would what you to be emptying it and storing it when it’s not being used” *– P10.*  “government organizations that sort of thing as well who are involved in safety around pools.” *– P14.* |
|  | Water safety advocates | “Laurie Lawrence, kids alive do the five, or whatever, that’s the only other water safety group that I can think about.” *– P03.* |
|  | Everyone and anyone | “everyone. Like I can’t think of a reason why you’d want just to leave a pool like just leave a risk lying around like a ticking time bomb.” *– P05.*  “Yes, most people would definitely approve of that.” *– P07.* |
|  | Friends | “Friends you know if they came over, they’d be expected to same sort of thing, they would not expect to have that outside.” *– P08.* |
|  |  |  |
| Disapprove |  |  |
|  | Water authorities and drought effected people | “Family who live, like they live in regional New South Wales at the moment and they’re in massive drought, so they probably wouldn’t be too impressed to be honest, but I think … And when I was living in those drought affected areas then obviously you’re more conscious of your water use whereas especially being up here in the territory where you have rain a good four or five months of the year constantly, I do not think about my water use as much at all. I’d say it would probably be people who could utilise the water way better than what we are.” *– P02.*  “Well I guess water-wise and the government wouldn’t approve of me filling up the pool and letting it out.” *– P09.*  “greenies perhaps, when it comes to conserving water, it could be seen as a waste of water, but that’s about all. When I say greenies, I’m talking about people who are environmentally friendly.” *– P13.* |
|  | No one would disapprove | “I can’t, no; I can’t imagine anybody who would be against doing that” *– P03.*  “Can’t think of anyone that would disapprove of doing it?” *– P11.* |
|  |  |  |
| **Control Beliefs** |  |  |
| Facilitators |  |  |
|  | Ease of draining, deflating and drying | “It would make it easier if there was a, sort of like a quick release water removal part, like the one we have its just got a small little plug so you have to wait a long time for the water to drain so if that part of the process happened a little bit faster it would make it easier.” – *P11*.  “ease of or accessibility to inflating it and deflating it.” *– P05*.  “Some kind of easy draining port, some way that I could easily just let the water out would be a big one for me because as I say the main issue that I face is getting it, getting the water out and packing it up, I always leave it for my husband. Yeah if there was an easy way to drain the water out.” *– P03.* |
|  | Size and design of the pool | “the pool design themselves so like ours is one that is completely inflatable, it can be inflated by one person. It can also be emptied and deflated by one person but it takes a little bit longer to do that but we have no metal poles or anything like that, that needs to be … You know it truly is a portable pool whereas ones that maybe need to be assembled and then with the liners that go through them I think it depends on the effort that you have to be put through.” *– P01.*  “The big pool that we predominately use for the kids is that it folds up into a box and then we put the box away, so that makes it massively easy. So, I guess the type of plastic that it is allows it to fold so that’s a massive thing.” *– P08.*  *“*it’s just the size really, the size and weight.” *– P13.* |
|  | Space to store it | “Space as well I suppose, we’re lucky enough that we have a backyard with a shed and those type of things so we can actually pull ours down and put it in the shed whereas if you don’t … And our clamshell goes behind the barbeque on our patio, so we have that space to be able to do that. So you know if you’ve got a smaller sort of house or only a courtyard it’s a little bit more difficult I think to pack it up and put it away without the space to do that.” *– P01.*    “Like obviously just making sure that you’ve got the room to do it, to store it practically somewhere where it can be put and is easy enough to get to the next time you need it.” *– P02.* |
| Barriers |  |  |
|  | Time consuming | “For the bigger one it’s generally that we have to wait sort of a good week for it to dry and wipe it down so that it doesn’t mould, because if it’s not completely dry it’ll just mould while it’s in storage. So that generally is a bit of a process because we wipe it down and then wait for it to dry and then we have to sort of stretch out the corners and make sure turn it inside out, wipe it down again and make sure it’s dry and then we get a towel and wipe it all out and fold it up. So getting it dry is definitely a pain in the neck.” – *P08.*  *“*Just the time consuming aspect of getting all the water out even when you use the drain the small drain at the bottom of it, if that doesn’t get the water out you need to get a bucket and bucket the rest of the water out till it’s light enough to be able to tip it on its side and tip the rest out, if I’m doing it by myself it can be very difficult cause its very heavy, so yeah that’s the hardest part and then I guess the air, just getting the air out that’s just time consuming you have to sort of squeeze all the air out until you are able to actually fold it up and pack it away.” *– P11.* |
|  | Slow to drain or no plug | “I would like a faster drain and a faster run off, yeah and I suppose if it’s a windy day it’s hard to get it to sit on the drying rack, it could get blown off.” *– P04.*  “um, probably the fact that it doesn’t have a plug, but really apart from that you just have to have a bit of patience.” *– P10.* |
|  | Heavy Weight | “it’s big bulky and cumbersome.” *– P15.*  “it’s a sheer size thing for me. It’d be about three metres long by a metre and a half wide I suppose, like it’s a decent size.” *– P03.* |
|  | No Barriers | “Nothing is really hard about it, we just kind of make sure all the water is out of it as much as we can dump out of it and then I guess you just kind of make sure it’s not going to be … if you put away in the corner where the rain won’t be able to get at it, less amount of debris in it. And then you just yes you’ve just got to make sure it’s not going to fly off the balcony which is a low chance but we just go put it in the corner, so it doesn’t get out.” *– P07.*  “nothing, there’s nothing difficult about it.” *– P13.* |

Figure captions

Supplementary Figure 1: Thematic Map – Supervising Within Arms’ Reach

Supplementary Figure 2: Thematic Map - Fencing Portable pools Deeper than 30cm.

Supplementary Figure 3: Thematic map – Emptying and Storing Portable Pools Safely After Use
